# Correlates of Resilience of Older People in Times of Crisis

**DOI:** 10.1093/geroni/igab046.2676

**Published:** 2021-12-17

**Authors:** Isabelle Albert, Martine Hoffmann, Elke Murdock, Josepha Nell, Anna Kornadt

**Affiliations:** 1 University of Luxembourg, Esch-sur-Alzette, Diekirch, Luxembourg; 2 RBS, Itzig, Diekirch, Luxembourg; 3 Luxembourg Ministry of Education, Children and Youth, Luxembourg, Diekirch, Luxembourg

## Abstract

Since the beginning of the Covid-19 pandemic, efforts have been made to shield older adults from exposure to the virus due to an age-related higher risk for severe health outcomes. While a reduction of in-person contacts was necessary in particular during the first months of the pandemic, concerns about the immediate and longer-term secondary effects of these measures on subjective well-being were raised. In the present study, we focused on self-reported resilience of older people in a longitudinal design to examine risk and protective factors in dealing with the restrictions. Data from independently living people aged 60+ in Luxembourg were collected via a telephone/online survey after the first lockdown in June (N = 611) and September/October 2020 (N = 523), just before the second pandemic wave made restrictions necessary again. Overall, results showed an increase in life-satisfaction from T1 to T2, although life-satisfaction was still rated slightly lower than before the crisis. Also, about a fifth of participants indicated at T2 difficulties to recover from the crisis. Participants who reported higher resilience to deal with the Covid-19 crisis at T2 showed higher self-efficacy, agreed more strongly with measures taken by the country and felt better informed about the virus. In contrast, participants who reported more difficulties in dealing with the pandemic, indicated reduced social contacts to family and friends at T2, and also felt lonelier. Results will be discussed applying a life-span developmental and systemic perspective on risk and protective factors in dealing with the secondary impacts of the pandemic.

